# Exploring the Impact of Batch Size on Deep Learning Artificial Intelligence Models for Malaria Detection

**DOI:** 10.7759/cureus.60224

**Published:** 2024-05-13

**Authors:** Rohit Muralidhar, Michelle L Demory, Marc M Kesselman

**Affiliations:** 1 Medicine, Nova Southeastern University Dr. Kiran C. Patel College of Osteopathic Medicine, Fort Lauderdale, USA; 2 Medical Education, Nova Southeastern University Dr. Kiran C. Patel College of Allopathic Medicine, Fort Lauderdale, USA; 3 Rheumatology, Nova Southeastern University Dr. Kiran C. Patel College of Osteopathic Medicine, Fort Lauderdale, USA

**Keywords:** medical innovation, public health, malaria screening, healthcare technology, artificial intelligence (ai)

## Abstract

Introduction

Malaria is a major public health concern, especially in developing countries. Malaria often presents with recurrent fever, malaise, and other nonspecific symptoms mistaken for influenza. Light microscopy of peripheral blood smears is considered the gold standard diagnostic test for malaria. Delays in malaria diagnosis can increase morbidity and mortality. Microscopy can be time-consuming and limited by skilled labor, infrastructure, and interobserver variability. Artificial intelligence (AI)-based tools for diagnostic screening can automate blood smear analysis without relying on a trained technician. Convolutional neural networks (CNN), deep learning neural networks that can identify visual patterns, are being explored for use in abnormality detection in medical images. A parameter that can be optimized in CNN models is the batch size or the number of images used during model training at once in one forward and backward pass. The choice of batch size in developing CNN-based malaria screening tools can affect model accuracy, training speed, and, ultimately, clinical usability. This study explores the impact of batch size on CNN model accuracy for malaria detection from thin blood smear images.

Methods

We used the publicly available “NIH-NLM-ThinBloodSmearsPf” dataset from the United States National Library of Medicine, consisting of blood smear images for Plasmodium falciparum. The collection consists of 13,779 “parasitized” and 13,779 “uninfected” single-cell images. We created four datasets containing all images, each with unique randomized subsets of images for model testing. Using Python, four identical 10-layer CNN models were developed and trained with varying batch sizes for 10 epochs against all datasets, resulting in 16 sets of outputs. Model prediction accuracy, training time, and F1-score, an accuracy metric used to quantify model performance, were collected.

Results

All models produced F1-scores of 94%-96%, with 10 of 16 instances producing F1-scores of 95%. After averaging all four dataset outputs by batch size, we observed that, as batch size increased from 16 to 128, the average combined false positives plus false negatives increased by 15.4% (130-150), and the average model F1-score accuracy decreased by 1% (95.3%-94.3%). The average training time also decreased by 28.11% (1,556-1,119 seconds).

Conclusion

In each dataset, we observe an approximately 1% decrease in F1-score as the batch size was increased. Clinically, a 1% deviation at the population level can create a relatively significant impact on outcomes. Results from this study suggest that smaller batch sizes could improve accuracy in models with similar layer complexity and datasets, potentially resulting in better clinical outcomes. Reduced memory requirement for training also means that model training can be achieved with more economical hardware. Our findings suggest that smaller batch sizes could be evaluated for improvements in accuracy to help develop an AI model that could screen thin blood smears for malaria.

## Introduction

Malaria is a major public health problem and a leading cause of death in developing countries worldwide [1]. According to the WHO, there were approximately 247 million cases of malaria in 2021 across 84 countries. Mortality has steadily decreased since 2000. However, in 2020, malaria cases resulting in death increased to 625,000 and remained at 619,000 in 2021. While malaria is a global disease, many cases can be localized to specific world regions because 29 of the 84 countries account for 96% of the cases globally. The WHO Africa region was estimated to account for 234 million of the 247 million cases globally in 2021 [1,2].

Unicellular protozoa of the Plasmodium genus are responsible for the pathogenesis of malaria. Some species of Plasmodium include Plasmodium falciparum, Plasmodium vivax, Plasmodium ovale, and Plasmodium knowlesi [3]. Transmission of Plasmodium in humans is mainly via a bite from a female Anopheles mosquito [4]. When an infected female Anopheles mosquito bites a human, sporozoites from its salivary glands enter the bloodstream [5]. Plasmodium parasites ultimately invade erythrocytes, where growth and replication occur [6].

Diagnostic tests for malaria

For a patient suspected of having malaria, the two diagnostic tests for confirmation are real-time diagnostic tests or light microscopy of peripheral blood smears, with the latter considered the gold standard for malaria diagnosis [7]. Two types of blood smears can be prepared: thin and thick blood smears. Thin and thick blood smears both involve a drop of blood on a slide, where the thin blood smear is spread into a thinner layer across a larger area and is fixed in methanol [8]. Thin and thick smears are subsequently stained with Giemsa stain before observation [7]. Through microscopic visualization, the species can be identified, infection staging can be assessed, and parasite density can be determined. The major disadvantages of microscopy-based diagnosis are the lack of available skilled technicians and infrastructure to support the preparation and analysis of blood smears [9]. Immunochromatographic rapid diagnostic tests (RDTs), which fall under the category of real-time diagnostic tests, on the other hand, test for antigens or enzymes specific to Plasmodium species. Antigenic testing for P. falciparum histidine-rich protein 2 (pfHRP2 ) is specific for P. falciparum, P. falciparum histidine-rich protein 2 (pLDH) can be specific for P. falciparum and P. vivax, and the aldolase enzyme is a pan-malarial antigen [7].

BinaxNOW is an FDA-approved RDT (BinaxNOW™, Abbott, Chicago, IL) that detects HRP2 and Aldolase to identify P. falciparum and generic Plasmodium, respectively. Data from a trial with BinaxNOW suggested an overall sensitivity of 82% for pan-Plasmodium detection and a sensitivity of 95% for P. falciparum [10]. In a different study with patients in Cameroon, SD Bioline, a histidine and LDH-based RDT, had an overall diagnostic sensitivity of 95.33%, with a specificity of 94.34%. In the same study, manual light microscopy-based diagnosis was determined to have a sensitivity of 94.86%, with a specificity of 94.34% as well. PCR detection of malaria was the control methodology to assess diagnostic accuracy [11]. In developing and evaluating new screening and diagnostic methodologies, these specificities and sensitivities can therefore be used as benchmarks.

The clinical presentation of malaria typically includes recurrent fever, headache, malaise, and muscular pains among other symptoms that can be mistaken for gastrointestinal infection or influenza [12]. Rapid diagnosis and treatment are therefore important in the management of malaria because delays in the diagnosis and treatment of malaria result in increased patient morbidity and mortality [13]. Delayed diagnosis and treatment of malaria can lead to serious complications such as cerebral malaria, renal failure, and pulmonary edema, elevating both mortality and morbidity risks [4]. Microscopy, the gold standard diagnostic modality, can be time-consuming, and, as discussed above, is limited by skilled labor and infrastructure. Additionally, inter-technician variability in the interpretation of blood smears impacts interobserver reliability across time and geography [14].

The role of artificial intelligence (AI) in diagnostic screening

AI-based tools for diagnostic screening allow for an automated and scalable approach to analyzing blood films without the need for a trained human microscopy interpreter [15]. Computer vision is a field within the broader AI umbrella that focuses on allowing computers to analyze and obtain information from images and videos [16]. In medicine, computer vision can be applied to various types of images to identify specific image features for diagnostic or screening purposes [17]. One key computer vision-based method is to use a convolutional neural network (CNN), which is a form of deep learning neural network architecture that can be used to identify visual patterns. CNNs attempt to learn features, including local relationships and patterns, about an image from its pixels and their relationship to one another. CNNs are thus being explored for their application in medical image analysis, specifically use cases such as abnormality detection and disease classification. CNNs can be used to explore different image modalities such as X-rays, MRIs, and CTs [18]. CNNs are also being applied to screen and identify pathology on blood films, such as the study conducted by Torres et al. in Peru that assessed the potential for a CNN-based malaria detection device [14].

Among the many parameters that can be optimized in CNN model building, one such hyperparameter is batch size, which is the number of images used during model training at once in one forward and backward pass [19]. The choice of batch size has a profound impact on model accuracy, the degree of overfitting, time to convergence, and training speed [20]. In addition to the quality of the trained model, batch size directly affects the amount of memory that the model requires during training because of the number of concurrent images that are fed into the model for training [21]. Thus, factors such as hardware constraints are considerations when selecting a batch size for a particular use case. Additionally, depending on the type of image dataset being used, there may be an optimal batch size to maximize model accuracy. At the intersection of optimizing for accuracy and working within hardware constraints sits a potential range of ideal batch size parameters for a particular problem. In this study, we develop a CNN model and train it on single-cell thin blood smear images. The AI model is similarly trying to identify Giemsa-stained blood smear images as would be analyzed in light microscopy. We then tested the model against different blood smear image datasets, with varying batch sizes to control for the impact that batch size may have on outputs such as model accuracy. In this study, we thus sought to explore the impact that batch size ultimately has on CNN model training with single-cell thin blood smear images via test accuracy and the potential clinical impact that these variations can have at scale.

## Materials and methods

Dataset description

The “NIH-NLM-ThinBloodSmearsPf” dataset, a publicly available dataset courtesy of the United States National Library of Medicine, consists of a set of blood smear images for P. falciparum. The dataset was developed by the National Library of Medicine of the National Institutes of Health. This dataset was originally used for cell detection in the publication by Yasmin et al. [22]. The dataset consists of thin blood smear microscopy images that were obtained originally from 193 patients (148 P. falciparum-infected and 45 uninfected control patients). Single-cell images from the microscopy images, categorized as “parasitized” and “uninfected,” were available for download and published on the National Library of Medicine Malaria data website (https://lhncbc.nlm.nih.gov/LHC-research/LHC-projects/image-processing/malaria-datasheet.html).

Per the dataset documentation, the microscopy images were obtained from patients at Chittagong Medical College Hospital in Bangladesh. Thin blood smears were Giemsa-stained, and images were captured using a smartphone camera. Images were then annotated and reviewed manually by an expert. The original study from which these images were captured was approved under the Institutional Review Board (IRB) at the National Library of Medicine, National Institutes of Health (IRB#12972).

**Figure 1:**
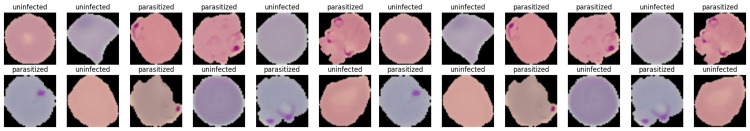
Random selection of images from the 13,779 parasitized and 13,779 unparasitized images, along with corresponding labels, after resizing and importing for use in models.

The single-cell image collection consists of 13,779 single-cell images categorized as “parasitized” and 13,779 categorized as “uninfected” (Figure 1).

Analysis tools

CNN models were developed with the Python programming language (Python version 3.10.12), which is an open-source language often used for data analysis and computing. Some of the Python libraries used in this study include pandas (v1.5.3), numpy (v 1.23.5), tensorflow (v2.13.0), and keras (v2.13.1). A complete list of Python libraries and versions is included in Appendix 1.

Google Colab was used as the development environment. Randomized data was stored on Google Drive for direct access from the Google Colab development environment. Outcome metrics were recorded in Microsoft Excel, and subsequent analysis was performed in Microsoft Excel. Additional analysis and visualization of data were done using the R programming language, which is an open-source programming language used for statistical analysis and data visualization. Analysis in R statistical software (version 2023.06.2+561; R Foundation for Statistical Computing, Vienna, Austria) was done using R studio.

Data randomization

The single-cell images were downloaded from the National Library of Medicine’s Malaria Data Website.

**Figure 2:**
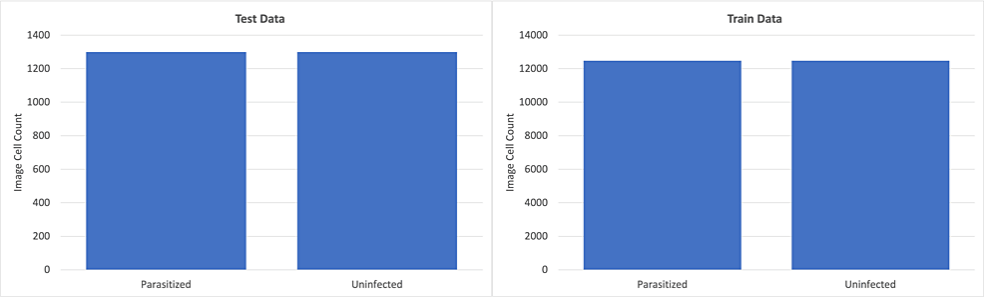
Left: number of parasitized (n = 1,300) and uninfected (n = 1,300) images set aside for model testing. Right: graphical depiction of the number of parasitized (n = 12,479) and uninfected (n = 12,479) images set aside for model training.

Using a Python script (Appendix 2), four different randomized datasets were created: Randomization_1, Randomization_2, Randomization_3, and Randomization_4. For each dataset, the script randomly selected 1,300 of the original images labeled as “parasitized” (P. falciparum-infected cells) and 1,300 of the images categorized as “uninfected” and delegated them into a “test” folder which would then be used to assess the performance of trained CNN models. The remaining images were saved into a “train” folder and used to train CNN models. The result was four datasets, each consisting of 2,600 “test” images (1,300 “parasitized” and 1,300 “uninfected”) and 24,958 “train” images (12,479 “parasitized” and 12,479 “uninfected”) (Figure 2).

Analysis

For each randomized dataset, images were imported from the corresponding zip file, resized to 64x64 images, and normalized by dividing by 255 prior to use in any of the models.

**Figure 3:**
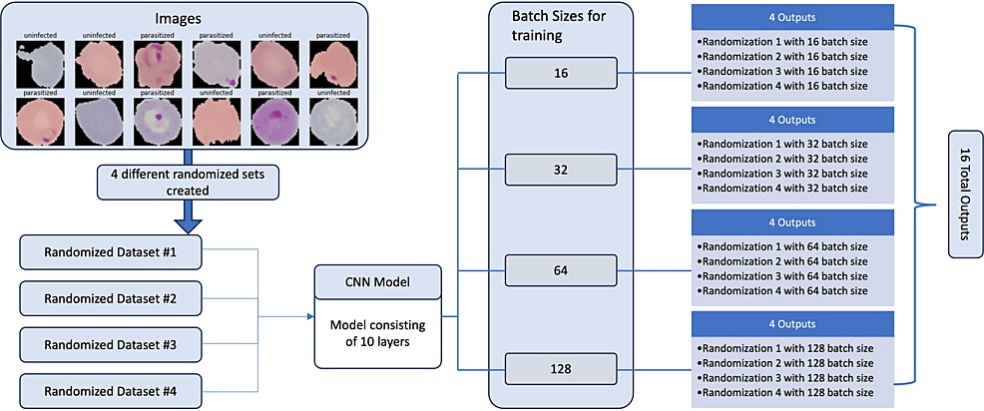
Overview of study design, depicting randomization of datasets, and the four specific batch sizes utilized in CNN models for each dataset.

Four identical 10-layer CNN models were created consisting of “Conv2D,” “MaxPooling2D,” “Dropout,” “Flatten,” and “Dense” layers. Each model was similarly trained with a validation split of 0.2, splitting 80% of the images set aside for training into actual training images and 20% into validation during model fitting. Models were fitted without callbacks and trained for 10 epochs. The differentiating feature of the four models was that they were trained with batch sizes of 16, 32, 64, and 128, respectively. These four models, parameterized with different batch sizes, were then trained and tested with the four randomized datasets prepared during data randomization, yielding a total of 16 sets of results iterated across the four models and four datasets (Figure 3).

For each of the 16 sets of model training and testing outputs, the following metrics were collected as data variables for analysis:

F1-score: The F1-score is an accuracy metric used to quantify the performance of binary medical tests and is the harmonic mean of precision and recall [23]. Mathematically, it can be calculated as follows:

F1-score = 2 x (Precision x Recall)/(Precision+Recall) [24].

True positive: Total number of instances when a CNN model correctly predicted “parasitized” from test images. This was computed and visualized using the “confusion_matrix()” Python script from the sklearn library.

True negative: Total number of instances when a CNN model correctly predicted “uninfected” from test images. This was computed using the “confusion_matrix()” Python script from the sklearn library.

False positive: Total number of instances when a CNN model incorrectly predicted “parasitized” from test images, when the image was actually classified as “uninfected.” This was computed using the “confusion_matrix()” Python script from the sklearn library.

False negative: Total number of instances when a CNN model incorrectly predicted “uninfected” from test images, when the image was actually classified as “parasitized.” This was computed using the “confusion_matrix()” Python script from the sklearn library.

Duration of training: The total amount of time, in seconds, taken for the model fitting to occur with training data, with 10 epochs. This was computed using the “confusion_matrix()” Python script from the sklearn library.

Training time: How long does the model take to complete training with 10 epochs.

In this study, when we refer to the “confusion matrix,” we are referring to the true-positive, true-negative, false-positive, and false-negative predictions by a CNN model.

The impact of batch size on model performance and clinical outcomes was assessed by comparing outcomes variables’ data when simulating against test images for each randomization and batch size combination. Comparative analysis and graphical interpretation of the data variables were conducted in Microsoft Excel and using R.

## Results

**Figure 4:**
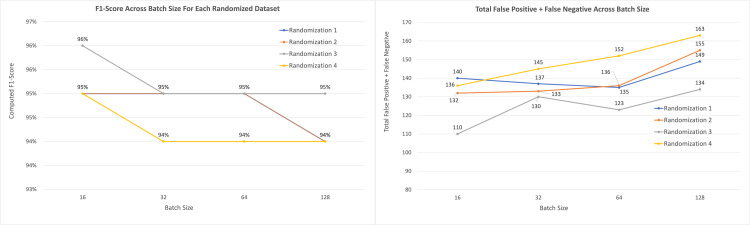
Left: F1-scores across batch sizes for each randomized dataset. Right: Total false-positive and false-negative CNN model predictions across batch size for each randomized dataset.

The four models with batch sizes of 16, 32, 64, and 128 were run against four sets of randomized data to produce 16 sets of output data. All the models produced F1-scores of 94% to 96%, with 10 of the 16 instances producing F1-scores of 0.95 and an average F1-score across all scenarios of 94.75% (Figure 4). The sum of the false negatives and false positives across all 16 instances ranged from 110 to 163, with a mean value of 138.125 (Figure 4).

After averaging all four dataset outputs by batch size, we observed that from batch sizes of 16-128, the average combined number of false positive plus false negative instances increased by 15.4% (130-150), which correlated with an overall model F1-score accuracy decrease of 1% (95.3%-94.3%). Training time also decreased by 28.11%, from 1,556 seconds to 1,119 seconds (Table 1).

**Table 1:** Confusion matrix, F1-score, and training time by batch size, after averaging results for randomized datasets 1-4.

	Confusion Matrix						
Batch Size	True Positive	True Negative	False Positive	False Negative	False Negative + False Positive	F1-Score Average	Training Time(s)
16	1,231	1,240	61	69	130	95.3%	1,556
32	1,230	1,234	66	71	136	94.8%	1,226
64	1,223	1,241	60	77	137	94.8%	1,092
128	1,234	1,216	84	67	150	94.3%	1,119

**Figure 5:**
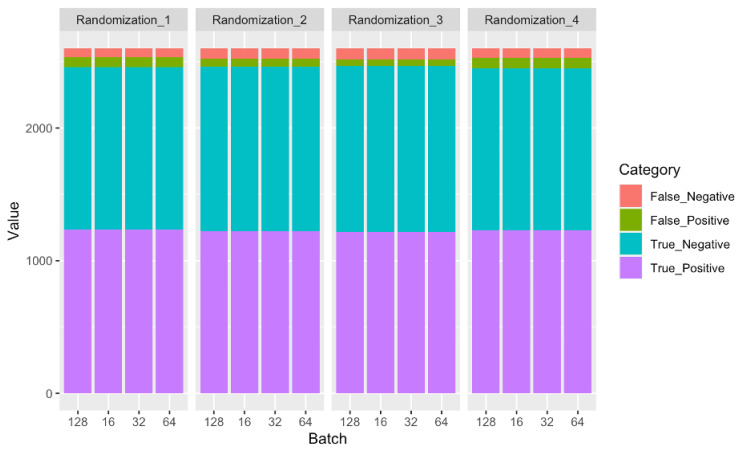
Stacked bar chart of confusion matrix data for each randomization vs. batch size combination analyzed in this study.

**Figure 6:**
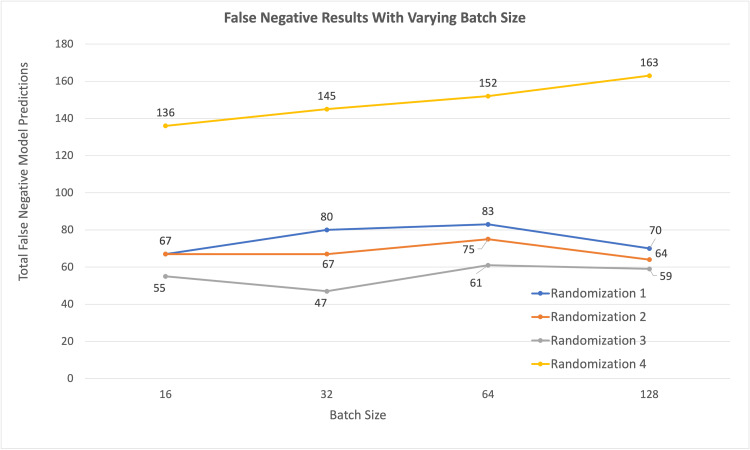
False-negative results for each dataset, with varying batch size.

However, it should be noted that average false negatives individually did not increase as steadily as the combined false negatives plus false positives, as batch size increased (Figures 5-6).

Table 2 presents a full summary of all model prediction accuracy variables that were calculated and training time for each randomization vs batch size CNN model analyzed in this study. The range (maximum minus minimum, or delta) for each metric by batch size was also calculated to analyze the dispersion of outcome data across randomized datasets and can be seen in Table 3.

**Table 2:** Full summary of positive, true negative, false positive, false negative, F1-score, and training time for each randomization dataset x batch size combination.

		Confusion Matrix						
Randomization Dataset	Batch Size						True Positive	True Negative	False Positive	False Negative	False Negative + False Positive	F1-Score Average	Training Time (s)
Randomization 1	16	1,233	1,227	73	67	140	95%	1,706
	32	1,220	1,243	57	80	137	95%	1,347
	64	1,217	1,248	52	83	135	95%	1,207
	128	1,230	1,221	79	70	149	94%	1,130
Randomization 2	16	1,233	1,235	65	67	132	95%	1,585
	32	1,233	1,234	66	67	133	95%	1,226
	64	1,225	1,239	61	75	136	95%	1,107
	128	1,236	1,209	91	64	155	94%	1,170
Randomization 3	16	1,245	1,245	55	55	110	96%	1,409
	32	1,253	1,217	83	47	130	95%	1,166
	64	1,239	1,238	62	61	123	95%	1,047
	128	1,241	1,225	75	59	134	95%	1,067
Randomization 4	16	1,213	1,251	49	87	136	95%	1,525
	32	1,212	1,243	57	88	145	94%	1,166
	64	1,211	1,237	63	89	152	94%	1,005
	128	1,227	1,210	90	73	163	94%	1,108

**Table 3:** Maximum-minimum value (delta, ∆) across the four randomized datasets was computed for each outcome metric by batch size.

	Confusion Matrix					
Batch Size	True Positive	True Negative	False Positive	False Negative	F1-Score Average	Training Time(s)
16	32	24	24	32	1.0%	297
32	41	26	26	41	1.0%	181
64	28	11	11	28	1.0%	202
128	14	16	16	14	1.0%	103

The false-negative deltas, or range of the observed false negatives, were 32, 41, 28, and 14 incorrect predictions for analyses run with batch sizes 16, 32, 64, and 128, respectively. Maximum value ranges varied by 58.2%, 87.2%, 45.9%, and 23.7% above the minimum values for each respective batch size. Notably, the dispersion metrics computed in Table 3, when compared to the total 2,600 predictions on test data for each randomized dataset made by models, varied by only small amounts. False-negative deltas computed in Table 3, for example, were only 1.2%, 1.6%, 1.1%, and 0.5% for batch sizes of 16, 32, 64, and 128, respectively. From Tables 2-3, we can see how the increase in incorrect model predictions with batch size is somewhat linear, although not specifically isolated to false-positive or false-negative predictions. It should be noted, however, from Table 3, that the dispersion of model prediction accuracy across datasets decreased as batch size increased, or the dispersion predictions, both false and true, from randomization one through four got smaller as batch size got larger. Another way to interpret this is that CNN models had more consistent results with different randomized isolated test images as batch size got larger.

**Figure 7:**
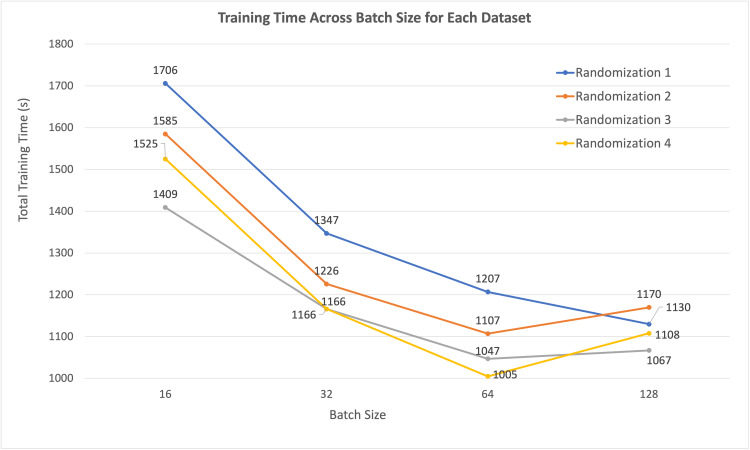
Total model training time for 10 epochs with varying batch size, for each dataset.

Total training time for model fitting with varying batch size, plotted for each dataset, demonstrated that training time also decreased as batch size increased (Figure 7). Ultimately, it took less time to iterate and train a model with the randomized blood smear datasets included in this study over 10 epochs as batch size increased.

## Discussion

Training a CNN model is an iterative statistical exercise, and many parameters can be adjusted in the hyper-optimization of a model. The goal in optimizing a model ultimately is to increase accuracy. Modifying batch size as a parameter not only affects performance but also affects the memory usage and training speed of a CNN. In this study, modifying batch size from 16 to 128 did not have a large impact mathematically on overall model accuracy. In all four datasets, we observed a 1% decrease in F1-score as the batch size was increased, with the Randomization_3 dropping from 96% to 95%, and the remaining three datasets dropping from 95% to 94%.

Clinically, however, a 1% deviation at the population level can make a relatively significant impact on outcomes. A 1% deviation across 1,000,000 patients, for example, can result in 10,000 people with incorrect screening results. A major goal of developing a malaria screening tool with a CNN is to introduce scalable screening in regions where malaria is endemic and access to physicians may be restricted. An applied solution leveraging AI for screening might include a process wherein local skilled labor can obtain blood samples, prepare slides, and upload the images to the cloud where a trained CNN model can screen in real-time whether it believes the sample to indicate that the patient is infected or uninfected. The implementation of such a solution reduces the need to locally maintain hardware for the model to run, which in some parts of the world may be costly and hard to do. If a screening tool is used to flag patients as at risk for being infected and speed up the time to diagnosis of malaria, false positives in a model can be managed by subsequently checking all positive screening blood smears manually. The false negatives, however, would fall through the gap as the screening tool would never indicate that the patient does in fact need treatment.

While the 16 sets of batch size vs. datasets that were run demonstrated an overall increase in CNN model accuracy and an increase in combined false positives and false negatives with decreasing batch size, the trend was less visible with just false-negative model predictions (Table 3).

According to Kandel et al.’s study exploring the effect of batch size on the generalizability of CNNs, they recommend using small batch sizes with low learning rates [19]. Additionally, Masters et al. in their study state that small batch sizes had the best generalization performance for a given computational cost [25].

Results from this study suggest that the improved accuracy with smaller batch sizes in models with similar layer complexity not only has better implications for clinical outcomes but also the reduced memory requirements for training means that model training can be achieved with more economical hardware. The increased training time that occurs with smaller batch sizes can make model development time-consuming and cumbersome, but this can be managed in clinical solutions by managing the trade-off with less frequent retraining of a model. Once a model is trained and implemented clinically, the subsequent retraining is something that needs to be managed over time. Retraining is the idea that as new images are collected over time and datasets get larger, models can be updated to reflect the more current or comprehensive datasets. While training or retraining a model with smaller batch sizes might take longer, once a model is trained the implemented solution and subsequent screening for each patient is very quick and rapid. It would typically just involve loading one or a few images into the trained model for assessment, so there is ultimately no increase in time for screening experienced by the patient.

Limitations of the study

Limitations of the study include the availability of diverse images, while there were a lot of images the data all came from one source and was already processed and cleaned, whereas clinically single-cell data might not be as quickly and easily available. This dataset also represented a relatively small number of patients overall. Additionally, in a clinical context, blood smear images that are most readily available would have many cells on display as opposed to single cells, which have to be localized on the image and cropped. Images with more cells may present a different set of optimization parameters for model development and may have varying results for accuracy.

Another limitation of the study was the hardware and software constraints. We used Google Colab as our environment for coding and analysis and were thus limited in computational resources available for free to use in the study. More powerful computers with dedicated GPUs could be useful in expanding the scope of the study and the complexity of the models used.

Implications for future research

Future research for CNN models needs to incorporate prospective real-time model performance in an applied clinical setting. Much of the CNN research that is being conducted is on retrospective datasets with images set aside for simulated real-time testing. The future of AI research needs to consider true prospective real-time performance to provide greater feedback on clinical applications and performance in a clinical setting. Training for these models should consider varying batch sizes for model optimization.

## Conclusions

The results from our study suggest that the increased optimization of the proposed model can be achieved when training with a batch size of 16 and that model accuracy may be increased with decreased batch size. Our findings thus suggest that smaller batch sizes could be evaluated for improvements in accuracy to help develop an AI model that could screen thin blood smears for malaria. In practice, smaller batch size training may be more feasible because of reduced hardware demands. The subsequent increase in model training time makes iterative training and retraining more cumbersome; however, as training can occur independently of screening, it should not affect screening speed for patients in practice.

## Appendices

Appendix 1: Python libraries

A comprehensive list of Python libraries and version numbers used in this study.

**Table 4:** Comprehensive list of Python libraries and version numbers used in this study.

Package	Version
absl-py	1.4.0
aiohttp	3.8.5
aiosignal	1.3.1
alabaster	0.7.13
albumentations	1.3.1
altair	4.2.2
anyio	3.7.1
appdirs	1.4.4
argon2-cffi	23.1.0
argon2-cffi-bindings	21.2.0
array-record	0.4.1
arviz	0.15.1
astropy	5.3.3
astunparse	1.6.3
async-timeout	4.0.3
attrs	23.1.0
audioread	3.0.0
autograd	1.6.2
Babel	2.12.1
backcall	0.2.0
beautifulsoup4	4.11.2
bleach	6.0.0
blinker	1.4
blis	0.7.10
blosc2	2.0.0
bokeh	3.2.2
bqplot	0.12.40
branca	0.6.0
build	1.0.3
CacheControl	0.13.1
cachetools	5.3.1
catalogue	2.0.9
certifi	2023.7.22
cffi	1.15.1
chardet	5.2.0
charset-normalizer	3.2.0
chex	0.1.7
click	8.1.7
click-plugins	1.1.1
cligj	0.7.2
cloudpickle	2.2.1
cmake	3.27.4.1
cmdstanpy	1.1.0
colorcet	3.0.1
colorlover	0.3.0
colour	0.1.5
community	1.0.0b1
confection	0.1.2
cons	0.4.6
contextlib2	21.6.0
contourpy	1.1.0
convertdate	2.4.0
cryptography	41.0.3
cufflinks	0.17.3
cvxopt	1.3.2
cvxpy	1.3.2
cycler	0.11.0
cymem	2.0.7
Cython	3.0.2
dask	2023.8.1
datascience	0.17.6
db-dtypes	1.1.1
dbus-python	1.2.18
debugpy	1.6.6
decorator	4.4.2
defusedxml	0.7.1
distributed	2023.8.1
distro	1.7.0
dlib	19.24.2
dm-tree	0.1.8
docutils	0.18.1
dopamine-rl	4.0.6
duckdb	0.8.1
earthengine-api	0.1.368
easydict	1.1
ecos	2.0.12
editdistance	0.6.2
eerepr	0.0.4
en-core-web-sm	3.6.0
entrypoints	0.4
ephem	4.1.4
et-xmlfile	1.1.0
etils	1.4.1
etuples	0.3.9
exceptiongroup	1.1.3
fastai	2.7.12
fastcore	1.5.29
fastdownload	0.0.7
fastjsonschema	2.18.0
fastprogress	1.0.3
fastrlock	0.8.2
filelock	3.12.2
Fiona	1.9.4.post1
firebase-admin	5.3.0
Flask	2.2.5
flatbuffers	23.5.26
flax	0.7.2
folium	0.14.0
fonttools	4.42.1
frozendict	2.3.8
frozenlist	1.4.0
fsspec	2023.6.0
future	0.18.3
gast	0.4.0
gcsfs	2023.6.0
GDAL	3.4.3
gdown	4.6.6
geemap	0.26.0
gensim	4.3.2
geocoder	1.38.1
geographiclib	2
geopandas	0.13.2
geopy	2.3.0
gin-config	0.5.0
glob2	0.7
google	2.0.3
google-api-core	2.11.1
google-api-python-client	2.84.0
google-auth	2.17.3
google-auth-httplib2	0.1.0
google-auth-oauthlib	1.0.0
google-cloud-bigquery	3.10.0
google-cloud-bigquery-connection	1.12.1
google-cloud-bigquery-storage	2.22.0
google-cloud-core	2.3.3
google-cloud-datastore	2.15.2
google-cloud-firestore	2.11.1
google-cloud-functions	1.13.2
google-cloud-language	2.9.1
google-cloud-storage	2.8.0
google-cloud-translate	3.11.3
google-colab	1.0.0
google-crc32c	1.5.0
google-pasta	0.2.0
google-resumable-media	2.6.0
googleapis-common-protos	1.60.0
googledrivedownloader	0.4
graphviz	0.20.1
greenlet	2.0.2
grpc-google-iam-v1	0.12.6
grpcio	1.57.0
grpcio-status	1.48.2
gspread	3.4.2
gspread-dataframe	3.3.1
gym	0.25.2
gym-notices	0.0.8
h5netcdf	1.2.0
h5py	3.9.0
holidays	0.32
holoviews	1.17.1
html5lib	1.1
httpimport	1.3.1
httplib2	0.22.0
humanize	4.7.0
hyperopt	0.2.7
idna	3.4
imageio	2.31.3
imageio-ffmpeg	0.4.8
imagesize	1.4.1
imbalanced-learn	0.10.1
imgaug	0.4.0
importlib-metadata	6.8.0
importlib-resources	6.0.1
imutils	0.5.4
inflect	7.0.0
iniconfig	2.0.0
intel-openmp	2023.2.0
ipyevents	2.0.2
ipyfilechooser	0.6.0
ipykernel	5.5.6
ipyleaflet	0.17.3
ipython	7.34.0
ipython-genutils	0.2.0
ipython-sql	0.5.0
ipytree	0.2.2
ipywidgets	7.7.1
itsdangerous	2.1.2
jax	0.4.14
jaxlib	0.4.14+cuda11.cudnn86
jeepney	0.7.1
jieba	0.42.1
Jinja2	3.1.2
joblib	1.3.2
jsonpickle	3.0.2
jsonschema	4.19.0
jsonschema-specifications	2023.7.1
jupyter-client	6.1.12
jupyter-console	6.1.0
jupyter_core	5.3.1
jupyter-server	1.24.0
jupyterlab-pygments	0.2.2
jupyterlab-widgets	3.0.8
kaggle	1.5.16
keras	2.13.1
keyring	23.5.0
kiwisolver	1.4.5
langcodes	3.3.0
launchpadlib	1.10.16
lazr.restfulclient	0.14.4
lazr.uri	1.0.6
lazy_loader	0.3
libclang	16.0.6
librosa	0.10.1
lightgbm	4.0.0
linkify-it-py	2.0.2
lit	16.0.6
llvmlite	0.39.1
locket	1.0.0
logical-unification	0.4.6
LunarCalendar	0.0.9
lxml	4.9.3
Markdown	3.4.4
markdown-it-py	3.0.0
MarkupSafe	2.1.3
matplotlib	3.7.1
matplotlib-inline	0.1.6
matplotlib-venn	0.11.9
mdit-py-plugins	0.4.0
mdurl	0.1.2
miniKanren	1.0.3
missingno	0.5.2
mistune	0.8.4
mizani	0.9.3
mkl	2023.2.0
ml-dtypes	0.2.0
mlxtend	0.22.0
more-itertools	10.1.0
moviepy	1.0.3
mpmath	1.3.0
msgpack	1.0.5
multidict	6.0.4
multipledispatch	1.0.0
multitasking	0.0.11
murmurhash	1.0.9
music21	9.1.0
natsort	8.4.0
nbclassic	1.0.0
nbclient	0.8.0
nbconvert	6.5.4
nbformat	5.9.2
nest-asyncio	1.5.7
networkx	3.1
nibabel	4.0.2
nltk	3.8.1
notebook	6.5.5
notebook_shim	0.2.3
numba	0.56.4
numexpr	2.8.5
numpy	1.23.5
oauth2client	4.1.3
oauthlib	3.2.2
opencv-contrib-python	4.8.0.76
opencv-python	4.8.0.76
opencv-python-headless	4.8.0.76
openpyxl	3.1.2
opt-einsum	3.3.0
optax	0.1.7
orbax-checkpoint	0.3.5
osqp	0.6.2.post8
packaging	23.1
pandas	1.5.3
pandas-datareader	0.10.0
pandas-gbq	0.17.9
pandocfilters	1.5.0
panel	1.2.2
param	1.13.0
parso	0.8.3
partd	1.4.0
pathlib	1.0.1
pathy	0.10.2
patsy	0.5.3
pexpect	4.8.0
pickleshare	0.7.5
Pillow	9.4.0
pip	23.1.2
pip-tools	6.13.0
platformdirs	3.10.0
plotly	5.15.0
plotnine	0.12.3
pluggy	1.3.0
polars	0.17.3
pooch	1.7.0
portpicker	1.5.2
prefetch-generator	1.0.3
preshed	3.0.8
prettytable	3.8.0
proglog	0.1.10
progressbar2	4.2.0
prometheus-client	0.17.1
promise	2.3
prompt-toolkit	3.0.39
prophet	1.1.4
proto-plus	1.22.3
protobuf	3.20.3
psutil	5.9.5
psycopg2	2.9.7
ptyprocess	0.7.0
py-cpuinfo	9.0.0
py4j	0.10.9.7
pyarrow	9.0.0
pyasn1	0.5.0
pyasn1-modules	0.3.0
pycocotools	2.0.7
pycparser	2.21
pyct	0.5.0
pydantic	1.10.12
pydata-google-auth	1.8.2
pydot	1.4.2
pydot-ng	2.0.0
pydotplus	2.0.2
PyDrive	1.3.1
PyDrive2	1.6.3
pyerfa	2.0.0.3
pygame	2.5.1
Pygments	2.16.1
PyGObject	3.42.1
PyJWT	2.3.0
pymc	5.7.2
PyMeeus	0.5.12
pymystem3	0.2.0
PyOpenGL	3.1.7
pyOpenSSL	23.2.0
pyparsing	3.1.1
pyperclip	1.8.2
pyproj	3.6.0
pyproject_hooks	1.0.0
pyshp	2.3.1
PySocks	1.7.1
pytensor	2.14.2
pytest	7.4.1
python-apt	0.0.0
python-box	7.1.1
python-dateutil	2.8.2
python-louvain	0.16
python-slugify	8.0.1
python-utils	3.7.0
pytz	2023.3.post1
pyviz_comms	3.0.0
PyWavelets	1.4.1
PyYAML	6.0.1
pyzmq	23.2.1
qdldl	0.1.7.post0
qudida	0.0.4
ratelim	0.1.6
referencing	0.30.2
regex	2023.6.3
requests	2.31.0
requests-oauthlib	1.3.1
requirements-parser	0.5.0
rich	13.5.2
rpds-py	0.10.2
rpy2	3.4.2
rsa	4.9
scikit-image	0.19.3
scikit-learn	1.2.2
scipy	1.11.2
scooby	0.7.2
scs	3.2.3
seaborn	0.12.2
SecretStorage	3.3.1
Send2Trash	1.8.2
setuptools	67.7.2
shapely	2.0.1
six	1.16.0
sklearn-pandas	2.2.0
smart-open	6.4.0
sniffio	1.3.0
snowballstemmer	2.2.0
sortedcontainers	2.4.0
soundfile	0.12.1
soupsieve	2.5
soxr	0.3.6
spacy	3.6.1
spacy-legacy	3.0.12
spacy-loggers	1.0.4
Sphinx	5.0.2
sphinxcontrib-applehelp	1.0.7
sphinxcontrib-devhelp	1.0.5
sphinxcontrib-htmlhelp	2.0.4
sphinxcontrib-jsmath	1.0.1
sphinxcontrib-qthelp	1.0.6
sphinxcontrib-serializinghtml	1.1.9
SQLAlchemy	2.0.20
sqlparse	0.4.4
srsly	2.4.7
statsmodels	0.14.0
sympy	1.12
tables	3.8.0
tabulate	0.9.0
tbb	2021.10.0
tblib	2.0.0
tenacity	8.2.3
tensorboard	2.13.0
tensorboard-data-server	0.7.1
tensorflow	2.13.0
tensorflow-datasets	4.9.2
tensorflow-estimator	2.13.0
tensorflow-gcs-config	2.13.0
tensorflow-hub	0.14.0
tensorflow-io-gcs-filesystem	0.33.0
tensorflow-metadata	1.14.0
tensorflow-probability	0.20.1
tensorstore	0.1.41
termcolor	2.3.0
terminado	0.17.1
text-unidecode	1.3
textblob	0.17.1
tf-slim	1.1.0
thinc	8.1.12
threadpoolctl	3.2.0
tifffile	2023.8.30
tinycss2	1.2.1
toml	0.10.2
tomli	2.0.1
toolz	0.12.0
torch	2.0.1+cu118
torchaudio	2.0.2+cu118
torchdata	0.6.1
torchsummary	1.5.1
torchtext	0.15.2
torchvision	0.15.2+cu118
tornado	6.3.2
tqdm	4.66.1
traitlets	5.7.1
traittypes	0.2.1
triton	2.0.0
tweepy	4.13.0
typer	0.9.0
types-setuptools	68.2.0.0
typing_extensions	4.5.0
tzlocal	5.0.1
uc-micro-py	1.0.2
uritemplate	4.1.1
urllib3	2.0.4
vega-datasets	0.9.0
wadllib	1.3.6
wasabi	1.1.2
wcwidth	0.2.6
webcolors	1.13
webencodings	0.5.1
websocket-client	1.6.2
Werkzeug	2.3.7
wheel	0.41.2
widgetsnbextension	3.6.5
wordcloud	1.9.2
wrapt	1.15.0
xarray	2023.7.0
xarray-einstats	0.6.0
xgboost	1.7.6
xlrd	2.0.1
xyzservices	2023.7.0
yarl	1.9.2
yellowbrick	1.5
yfinance	0.2.28
zict	3.0.0
zipp	3.16.2

Appendix 2: Randomization Python scripts

**Table 5:** Randomization scripts.

Parasitized Randomized Selection of Test Images:
import numpy as np
import os
import random
#set directories
directory = str('/Users/rohitmuralidhar/Desktop/Research_Data/Cell_Randomization_4/Parasitized')
target_directory = str('/Users/rohitmuralidhar/Desktop/Research_Data/Cell_Randomization_4/ParasitizedTEST')
# list all files in dir that are an image
files = [f for f in os.listdir(directory) if f.endswith('.png')]
# select a specified number of files randomly
random_files = random.sample(files, int(1300))
# move the randomly selected images by renaming directory
for random_file_name in random_files:
os.rename(directory+'/'+random_file_name, target_directory+'/'+random_file_name)
continue
Uninfected Randomized Selection of Test Images:
import numpy as np
import os
import random
#set directories
directory = str('/Users/rohitmuralidhar/Desktop/Research_Data/Cell_Randomization_4/Uninfected')
target_directory = str('/Users/rohitmuralidhar/Desktop/Research_Data/Cell_Randomization_4/UninfectedTEST')
# list all files in dir that are an image
files = [f for f in os.listdir(directory) if f.endswith('.png')]
# select a specified number of files randomly
random_files = random.sample(files, int(1300))
# move the randomly selected images by renaming directory
for random_file_name in random_files:
os.rename(directory+'/'+random_file_name, target_directory+'/'+random_file_name)
continue
